# The significance of decompressive craniectomy for older patients with traumatic brain injury: a propensity score matching analysis from large multi-center data

**DOI:** 10.1038/s41598-023-37283-x

**Published:** 2023-06-28

**Authors:** Jang Hun Kim, Kyung-Jae Park, Shin-Hyuk Kang, Dong-Hyuk Park, Jong Hyun Kim

**Affiliations:** 1 Department of Neurosurgery, Korea University Anam Hospital, Korea University College of Medicine, Seoul, Korea; 2 Department of Neurosurgery, Korea University Guro Hospital, Korea University College of Medicine, Seoul, Korea

**Keywords:** Neuroscience, Neurology

## Abstract

The efficacy of decompressive craniectomy (DC) for traumatic brain injury (TBI) have been investigated in two recent randomized clinical trials (RCTs) and DC is recommended as an optional treatment for improving overall survival compared to medical treatment. However, the two RCTs enrolled extremely young adults, and the efficacy of DC in older adults remains questionable. Therefore, to identify the efficacy of DC in older adults, we compared patients who received medical care with those who underwent DC after propensity score matching (PSM). From the Korea Multi-center Traumatic Brain Injury Database, 443 patients identified as having intracranial hypertension and a necessity of DC were retrospectively enrolled. The patients were classified into the DC (n = 375) and non-DC (n = 68) groups according to operation records. The PSM was conducted to match the patients in the DC group with those receiving medical care (non-DC). After PSM, the newly matched group (DC, n = 126) was compared with patients without DC (non-DC, n = 63). The mean difference in the logit of the propensity scores (LPS) was 0.00391 and the mean age of enrolled patients were 65 years. The results of the comparative analyses after PSM showed that the 6-month mortality rate of the non-DC group was higher than that of the DC group (61.9% vs. 51.6%, p = 0.179). In terms of favorable outcomes (modified Rankin Scale [mRS] score < 4), the DC group showed a lower rate of favorable mRS scores (11.9% vs. 17.5%, p = 0.296) than the non-DC group.

## Introduction

Decompressive craniectomy (DC) is a last-tier surgical intervention for lowering the intracranial pressure (ICP) in patients with severe traumatic brain injury (TBI), in which a large section of the skull is removed and the underlying dura mater is released^1^. Despite controversies, the use of DC has been introduced as a level IIA recommendation in a recent TBI guideline^2^ and its efficacy has been considered to be helpful in reducing the ICP and duration of intensive care and improving overall survival compared to those with medical treatment^3,4^. However, two recent randomized clinical trials (RCTs) that investigated the efficacy and safety of DC for TBI enrolled patients aged between 15 and 59 years and 10 and 65 years, respectively, and excluded older adults aged over 65 years. In addition, the mean ages of the enrolled patients in the two RCTs were 24 and 33 years, respectively. Therefore, these trials fail to represent the middle-aged patients as well.

In clinical settings, physicians usually perform DC when the monitored ICP is high and refractory despite the best medical care or when the estimated ICP is extremely high according to several radiologic (midline shifting or basal cistern collapse) and clinical findings (unresponsive pupils or low Glasgow coma scale [GCS] scores)^5^. In this situation, patients’ relatives or legal guardians should be thoroughly informed about the implications of DC to decide whether to perform it. Typically, patients who do not undergo DC are relatively older than those enrolled in the above RCTs, owing to which they may have comorbidities suspected to be associated with a poor prognosis^6,7^.

Using the multi-center TBI data (Korea), we were able to find patients who did not undergo DC due to several reasons. Because the patients who receive medical care are usually older than the patients of previous studies, we compared these patients with those who underwent DC for identifying the efficacy of DC in older patients with comorbidities. For reducing the bias of confounding variables, we compared these two groups after conducting a propensity score matching (PSM) analysis.

## Materials and methods

### Patients and data acquisition

In the Korea Multi-center TBI (KMTBI) databank, data from 4628 TBI patients were registered from 10 institutions (January 2016 to December 2018)^8^. Among them, 1167 patients with moderate-to-severe TBI (GCS < 13) were selected, and their medical records were reviewed. Finally, 443 patients identified as having intracranial hypertension and a necessity of DC were enrolled. The necessity of DC was defined as having (1) a refractory high monitored ICP (> 20 mmHg) despite the best neurocritical care or (2) the highly estimated ICP based on radiologic (basal cistern collapse or uncal herniation) and clinical findings (bilateral fixed dilated pupils or low GCS scores)^9^. The data that support the findings of this study are available from KMTBI investigators, but restrictions apply to the availability of these data, which were used under license for the current study, and so are not publicly available. Data are however available from the authors upon reasonable request and with permission of KMTBI investigators.

After patient enrollment, clinical data were acquired by reviewing the medical records. The case report form includes patient information (age, sex, past medical history of Charlson comorbidity index [CCI]^10^, and habitual and medication history), trauma mechanism, initial vital signs (heart rate [HR], respiration rate [RR], blood pressure [BP], body temperature, and oxygen saturation [SaO_2_]), initial neurologic status (GCS score, pupillary response, and level of consciousness), laboratory findings (blood cell counts, coagulation tests, renal and hepatic function test, and blood glucose and C-reactive protein levels), and their clinical courses. The outcome was measured by death at two weeks and the modified Rankin Scale (mRS) score at six months. In particular, radiological data from initial brain computed tomography (CT) scans were also registered and reviewed by two independent neurosurgeons in terms of the main diagnosis, degree of basal cistern compression and midline shifting, presence of epidural hematoma, subarachnoid hemorrhage, and intraventricular hemorrhage, and the Rotterdam CT score^11^. Discrepancies between the two reviewers were resolved after careful discussion. The current study design was approved by the institutional review board of the Human Research Center of Korea University Anam Hospital (K2020-1047-002), and the requirement for informed consent was waived owing to its retrospective design. The authors are able to confirm that all experiments were performed in accordance with relevant guidelines and regulations.

### Propensity score matching

**Figure 1:**
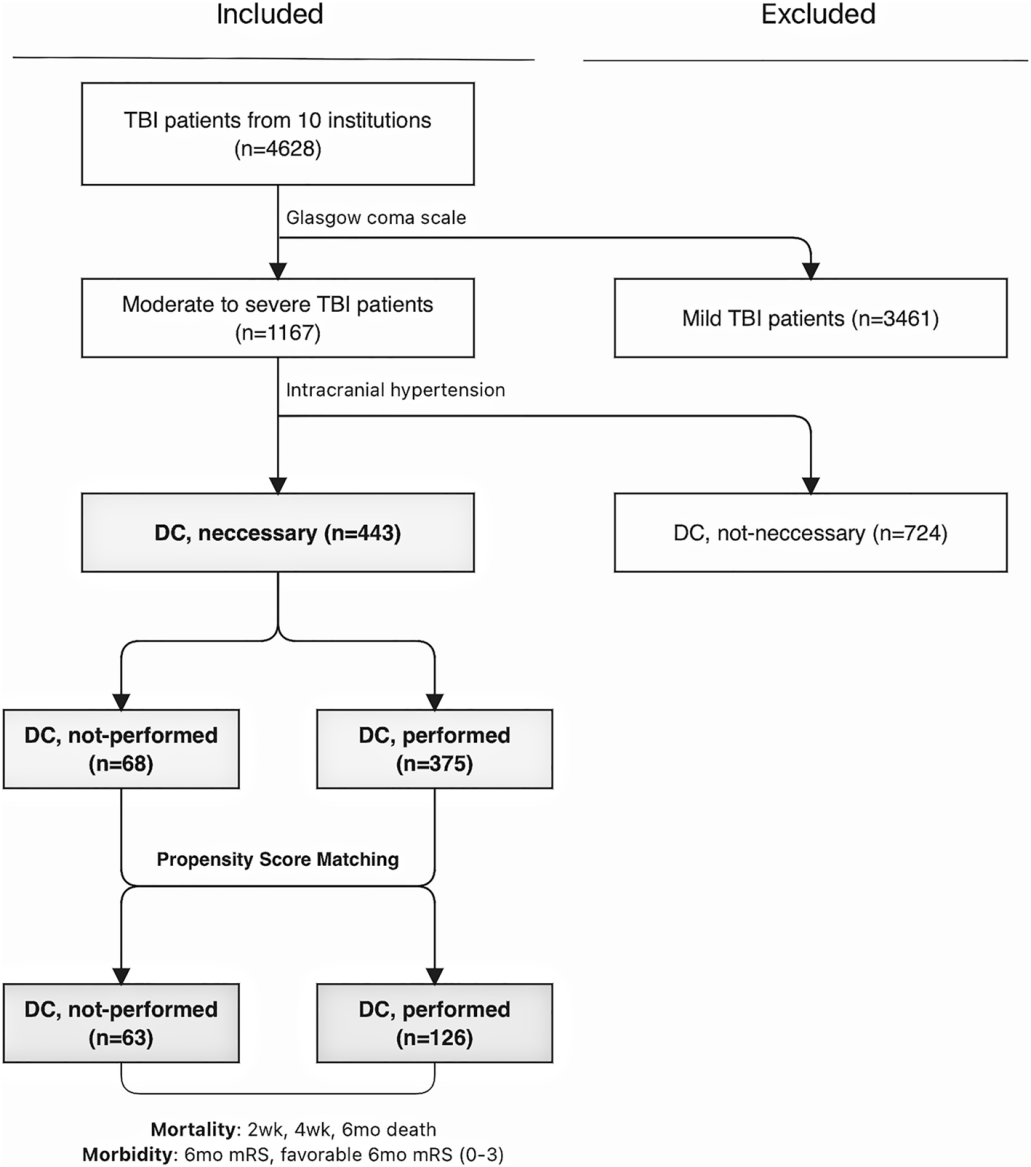
The flowchart of patient sorting and enrollment from the multi-centered TBI databank.

Before PSM, patients were classified into DC (n = 375) and non-DC (n = 68) groups based on the presence of operation records of DC and comparative analyses were performed. For PSM, logistic regression analysis was first conducted to estimate the correlation of each variable with the selected treatment (DC). The coefficient estimates from this regression were then used to retrospectively calculate a predicted probability, ranging from 0 to 1, of the treatment for each subject based on the individual’s specific characteristics. Each patient from the non-DC group was then matched with two patients from the control group based on the closest probability of treatment and the size of the available control group. Once each treatment subject was matched, the unused controls were removed, and an analysis was performed to compare DC outcomes with those of the newly matched control group (63 non-DC vs. 126 DC)^12^. A flowchart of patient classification and matched classification after PSM is shown in Fig. 1.

### Statistical analysis

Continuous values were presented as means and standard deviations, and categorical variable data were presented as numbers and percentages. Comparative analyses were performed between the two groups (DC vs. non-DC) before and after PSM. Additionally, comparison analyses between young (age < 65-year-old) and old groups (age ≥ 65-year-old) were conducted for identifying the differences between the groups. Independent t-tests or Mann–Whitney U tests were run on continuous variables according to the normal distributions, and *chi-square* tests on categorical variables. If more than 20% of the cells in the chi-square had an expected frequency of less than 5, the likelihood ratio or Fisher’s exact test was used. The statistical significance was set at p < 0.05. Statistical analyses were performed using standard software (version 23.0, SPSS, IBM, Chicago, IL, USA).

## Results

The results of the comparative analysis between the DC (n = 375) and non-DC (n = 68) groups before PSM are shown in the Supplementary Table 1. Several baseline parameters were significantly different, including age, CCI, RR, SaO_2_, and hypoxic events. The results of essential parameters are presented in Table 1. The DC group showed significantly lower mortality (51.2% vs. 64.7%) and better mRS scores (4.77 ± 1.714 vs. 5.19 ± 1.385).

**Table 1:** Differences in outcome parameters between the two groups (no-DC vs. DC).

Parameters	All (n = 443)		No DC (n = 68)		DC (n = 375)		Odds ratio	95% Confidence interval		p-value
Death at 2 weeks	193	(43.6%)	30	(44.1%)	163	(43.5%)	0.974	0.579	1.639	0.921
Death at 6 months	236	(53.3%)	44	(64.7%)	192	(51.2%)	0.572	0.334	0.979	0.040*
mRS at 6 months	4.83	± 1.673	5.19	± 1.385	4.77	± 1.714	0.423	-0.009	0.855	0.055
0	6	(1.4%)	0	(0.0%)	6	(1.6%)				0.028*
1	33	(7.4%)	1	(1.5%)	32	(8.5%)				
2	26	(5.9%)	6	(8.8%)	20	(5.3%)				
3	16	(3.6%)	4	(5.9%)	12	(3.2%)				
4	38	(8.6%)	1	(1.5%)	37	(9.9%)				
5	88	(19.9%)	12	(17.6%)	76	(20.3%)				
6	236	(53.3%)	44	(64.7%)	192	(51.2%)				
Favorable mRS score at 6 months	81	(18.3%)	11	(16.2%)	70	(18.7%)	0.841	0.419	1.686	0.621

*DC* decompressive craniectomy, *mRS* modified Rankin Scale.

**Figure 2:**
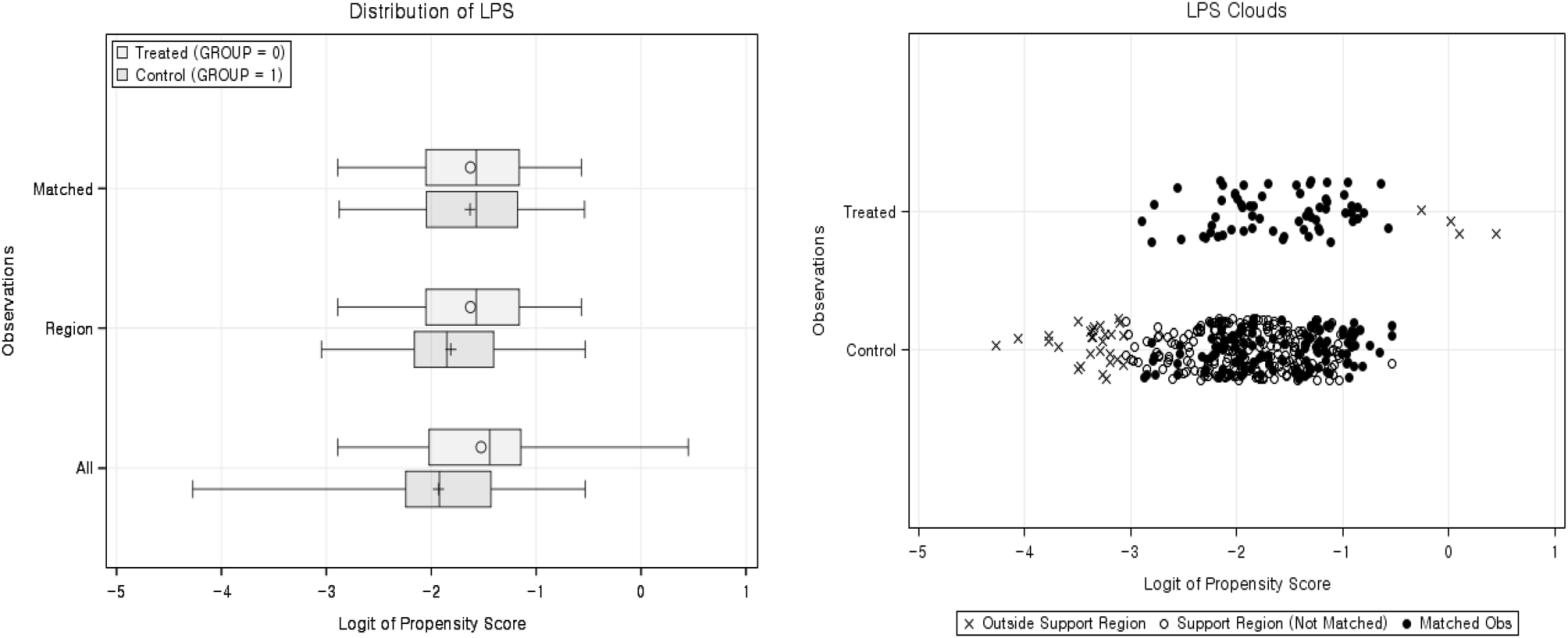
Plots of the logit of propensity score (LPS) distribution and clouds.

The results of the stepwise selection after the univariate logistic regression analyses are presented in Table 2. In the univariate analysis, parameters of age, CCI, HR, RR, and blood urea nitrogen were found to be significantly different between the groups. After stepwise selection, three parameters (age, HR, and RR) were selected. The PSM (1:2 matching) was conducted using the three selected parameters. The results of the PSM analysis are presented in Table 3 and Fig. 2. The mean difference in the LPS after matching was 0.00391, indicating that adequate matching was performed.

**Table 2:** Baseline differences between the two groups (no-DC vs. DC).

Parameters	All (n = 443)		No DC (n = 68)		DC (n = 375)		p-value (univariate LR)	p-value (stepwise selection)
Age	58.973	± 17.716	66.382	± 15.418	57.629	± 17.791	0.000*	0.0318*
CCI	0.666	± 1.254	0.971	± 1.820	0.611	± 1.115	0.034*	
GCS	7.799	± 3.776	7.106	± 3.534	7.924	± 3.811	0.173	
NBP	103.184	± 24.262	99.597	± 30.436	103.828	± 22.969	0.188	
HR	87.079	± 23.380	91.955	± 29.581	86.208	± 22.026	0.065	0.0105*
RR	19.238	± 4.215	18.299	± 5.710	19.405	± 3.874	0.049*	0.0265*
SaO_2_	94.652	± 7.789	92.391	± 12.252	95.066	± 6.598	0.019*	
Rotterdam score	4.178	± 1.338	4.132	± 1.434	4.187	± 1.321	0.758	
Hemoglobin	12.902	± 2.262	12.844	± 2.362	12.913	± 2.246	0.817	
Platelet	201.248	± 73.452	205.397	± 76.406	200.496	± 72.983	0.612	
INR	1.161	± 0.393	1.226	± 0.538	1.149	± 0.361	0.149	
BUN	17.270	± 10.123	15.437	± 5.192	17.602	± 10.750	0.091	

*LR* logistic regression analysis, *CCI* Charlson comorbidity index, *GCS* Glasgow coma scale, *NBP* non-invasive blood pressure, *HR* heart rate, *RR* respiratory rate, *INR* international normalized ratio, *BUN* blood urea nitrogen.

**Table 3:** Results of PSM analysis.

Variable	LPS
Mean difference	
All Obs	0.40583
Region Obs	0.186347
Matched Obs	0.002639
Standardized mean difference	
Divisor	0.67433
Mean difference	
All Obs	0.601826
Region Obs	0.276344
Matched Obs	**0.003914**
Percent reduction	
Region Obs	54.08
Matched Obs	99.35
Variance ratio	
All Obs	1.0769
Region Obs	1.1432
Matched Obs	1.0258

Significant values are in bold.

**Figure 3:**
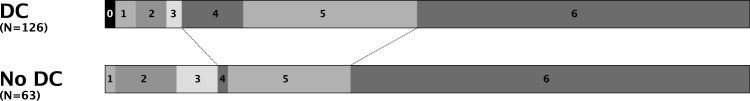
The distribution of the 6-month Modified Rankin Scale (mRS) scores of enrolled patients.

The results of the comparative analyses after PSM are presented in Table 4. No significant differences in the baseline characteristics were observed. In terms of outcome, the 6-month mortality rate of the DC group was lower than that of the non-DC group (51.6% vs. 61.9%); however, this difference was not significant (p = 0.179). The 6-month mRS scores are shown in Fig. 3. In terms of favorable outcomes (mRS score < 4), the DC group showed a lower rate of favorable mRS scores (11.9% vs. 17.5%) than the DC group; however, this difference was not significant (p = 0.296).

**Table 4:** Comparison after PSM (with age, HR, and RR parameters).

Baseline parameters	No-DC (n = 63)		DC (n = 126)		Mean difference or Odds ratio	95% confidential index		p-value
Age	65.54	± 15.570	64.67	± 14.880	0.873	− 3.727	5.473	0.709
Sex (male)	50	(79.4%)	93	(73.8%)	1.365	0.659	2.826	0.401
CCI	0.97	± 1.866	0.68	± 1.191	0.286	− 0.156	0.727	0.203
Mental Status								0.842
Alert	3	(4.8%)	6	(4.8%)				
Drowsy	8	(12.9%)	25	(19.8%)				
Stupor	22	(35.5%)	42	(33.3%)				
Semicoma	21	(33.9%)	38	(30.2%)				
Coma	8	(12.9%)	15	(11.9%)				
Pupil								0.468
Equal Reacting	24	(40.7%)	52	(45.6%)				
Unequal Reacting	4	(6.8%)	3	(2.6%)				
Only One Reacting	2	(3.4%)	7	(6.1%)				
Neither Reacting	29	(49.2%)	52	(45.6%)				
GCS score	7.57	± 3.451	8.03	± 4.007	− 0.462	− 1.882	0.958	0.521
NBP	102.37	± 29.121	102.19	± 27.827	0.175	− 8.429	8.778	0.968
HR	89.40	± 26.421	90.67	± 23.884	− 1.270	− 8.805	6.265	0.740
RR	19.21	± 4.194	18.99	± 3.787	0.214	− 0.981	1.410	0.724
SpO_2_	92.34	± 12.443	95.10	± 7.192	− 2.759	− 5.672	0.154	0.063
Rotterdam CT score	4.05	± 1.442	4.25	± 1.288	− 0.198	− 0.607	0.210	0.339
Hb	13.076	± 2.2489	12.805	± 2.2865	0.271	− 0.421	0.964	0.440
Platelet	202.33	± 76.282	194.85	± 74.347	7.484	− 15.344	30.312	0.519
INR	1.1930	± 0.49807	1.1464	± 0.30905	0.047	− 0.070	0.163	0.431
BUN	15.376	± 5.1818	18.722	± 11.6373	− 3.345	− 6.381	− 0.310	0.031
**Outcome parameters**								
Death_2wk	27	(42.9%)	57	(45.2%)	1.101	0.598	2.027	0.756
Death_1mo	33	(52.4%)	60	(47.6%)	0.826	0.451	1.514	0.537
Death_6mo	39	(61.9%)	65	(51.6%)	0.656	0.354	1.215	0.179
mRS_6mo	5.13	± 1.420	5.02	± 1.434	0.103	− 0.332	0.538	0.640
0	0	(0.0%)	2	(1.6%)				0.108
1	1	(1.6%)	4	(3.2%)				
2	6	(9.5%)	6	(4.8%)				
3	4	(6.3%)	3	(2.4%)				
4	1	(1.6%)	12	(9.5%)				
5	12	(19.0%)	34	(27.0%)				
6	39	(61.9%)	65	(51.6%)				
Favorable mRS_6mo (0–3)	11	(17.5%)	15	(11.9%)	1.565	0.673	3.644	0.296

The results of the comparative analyses between young (n = 269) and old groups (n = 174) were presented in Supplementary Table 2. Only the parameters of Charlson comorbidity index and SaO2 were significantly different. In terms of outcome, there was no significantly difference between the groups.

## Discussion

In this study, moderate-to-severe TBI patients who did not undergo DC were compared to those who underwent DC after PSM analysis. The patients enrolled in this study were older than those in the two previously reported RCTs, and the results showed an approximately 10% decrease in 6-month mortality in patients who underwent DC; however, they did not show favorable mRS scores. The current study used nationwide TBI data collected from 10 hospitals. This is the first subgroup analysis that has investigated the implications of DC using large multi-center data.

With the recent improvement of ICP monitoring techniques and widespread adoption of therapies to reduce the ICP^13–15^, DC can be allowed as a second-tier therapy in selected cases with brain edema not responsive to medical treatment^2,16^. The two recent investigations, named the DECRA and RESCUEicp trials^3,4^, organized a well-designed prospective randomized study; however, the enrolled patients were extremely young and did not represent middle-aged adults. In particular, these studies cannot be applied to older patients because they typically have other underlying medical conditions and are expected to have poorer prognoses than the younger patients^17,18^. Given this, physicians have difficulties making the decision to perform DC when they encounter a severe TBI patient of middle-to-old age.

In the current study, we focused on older adults (mean age, 65 years) who are expected to involve clinically dilemmatic cases. To acquire the most powerful evidence from this retrospective observational study, we designed a comparative analysis after adequate PSM. The PSM is an ideal technique that attempts to estimate the effect of a treatment by accounting for covariates related to the treatment. It attempts to reduce bias due to confounding variables in retrospective observational settings^12^. In this method, unused control units are naturally removed, and this may lead to a failure to secure an adequate number of patients for comparison. However, in our study, we retrospectively collected large amounts of data from multiple centers to conduct PSM analysis. We first selected the patients who did not undergo DC from a large multi-center database and matched them to patients who underwent DC. Naturally, the patients in the non-DC group were older and had comorbidities, and were significantly different from the DC group in these respects (Table 2). By comparing the groups after PSM, conclusions on the implications of DC were drawn.

Table 1 shows the masked results of DC, as similarly shown in a typical observational study. Before PSM, patients in the DC group showed a lower mortality rate at six months (51.2% vs. 64.7%) and slightly better functional outcomes (favorable mRS scores: 18.7% vs. 16.2%). Based on these results, DC can be recommended as an essential intervention for survival, because it does not have the adverse effect of poor functional outcomes. However, one criticism of these results may be that there was a bias in the patient enrollment into the two groups. The baseline difference can mask the unexpected effects and complications of DC and consequently, DC seems to be superior to medical care, as reported in several retrospective studies^19,20^. In clinical settings, the relatives or legal guardians of the patients are affected by the information (especially, ‘warnings’) given by the medical team. Therefore, patients chosen to receive medical care are usually older, have more extensive medical history, and are expected to have poor functional outcomes^17^. The baseline differences are shown in Table 2, including age, HR, and RR in the stepwise selection. Furthermore, the CCI was identified to be significant in the univariate logistic regression analysis. These results indicate that relatives or legal guardians of the patient typically decide to not choose DC when the patient has factors thought to be associated with poor outcomes. In addition, the patients receiving medical care are suspected to have other organ damage and poor vital signs (HR, RR, and SaO_2_). DC can be masked as being superior to medical therapy because previous studies do not correct these factors.

In the current study, we first set the control group as patients who had not received DC. As previously mentioned, they are older adults with extensive medical history and are expected to have poor outcomes. By performing PSM, we can indirectly infer their clinical courses and outcomes that may have resulted if they had received DC. Due to the underlying conditions, the 6-month mortality of the patients (non-DC: 61.9% and DC: 51.6%) was higher than that reported in prior RCTs. According to our results, if we performed DC in patients with these specific conditions, a survival rate of approximately 10% can be achieved (mortality: 48.4%– > 38.1%). However, in terms of functional outcomes, favorable mRS scores were lower in the DC group (no-DC: 17.5% and DC: 11.9%); and this difference was not significant. The proportion of mRS scores of 4 and 5 in the DC group was much larger than that in the non-DC group (Fig. 3). This result is similar to those from prior RCTs that reported that when patients received DC, their outcomes usually shifted from “both death and favorable outcomes” to “severe disability”. Our results suggest that physicians should inform the patients’ relatives or legal guardians that DC can increase the chances of survival of the patient (by approximately 10%); however, the patients have a higher chance of severe disability or the so-called “bed-ridden status”.

In clinical settings, the clinical course and outcomes of patients with severe TBI are extremely difficult to predict because they can be affected by multiple factors such as age, comorbidity, presence of vital organ damage, and accompanying therapeutic modality for reducing the ICP^13,21,22^ To determine the clinical efficacy and safety of DC, these factors should be strictly standardized. Despite prior investigators’ efforts to reduce errors, differences occur in the baseline characteristics and perioperative treatment that can affect the results of the study. In the current study, we conducted PSM to identify the true efficacy of DC using data from a large multi-center cohort. Fortunately, we were able to enroll patients far older than those in prior RCTs and our results are similar to those from prior RCTs. In conclusion, the results from RCTs (10–20% mortality reduction though favorable outcomes were not better) can be applied from younger to older patients.

The current study has several limitations. First, because the data were extracted from a multi-center retrospective databank, the perioperative protocols were not standardized between the different centers. Second, our results cannot be generalized and should be regarded only as a “reference” because of the small number of enrolled patients and the lack of a prospective design. Third, the cost-effectiveness of DC in terms of its associated intensive care unit and hospital stays was not evaluated.

## Conclusion

The current study performed a comparison of patients with moderate-to-severe TBI who underwent DC with those who received medical care, after PSM. The enrolled patients were significantly older than those in the previous two RCTs (mean age, 65 years) and an approximately 10% decrease in 6-month mortality was observed by performing DC; however, the mRS scores were not favorable.

## Supplementary Information

Supplementary Table 1.

Supplementary Table 2.

## Data Availability

The data that support the findings of this study are available from KMTBI investigators, but restrictions apply to the availability of these data, which were used under license for the current study, and so are not publicly available. Data are however available from the authors upon reasonable request and with permission of KMTBI investigators.
