# Supplementary material for: The significance of decompressive craniectomy for older patients with traumatic brain injury: a propensity score matching analysis from large multi-center data
Source: Sci Rep. 2023 Jun 28;13:10498. doi: 10.1038/s41598-023-37283-x (PMC10307774; doi:10.1038/s41598-023-37283-x)
Supplement: Supplementary file 1 — Supplementary Table 1. [file 41598_2023_37283_MOESM1_ESM.pdf]

Supplementary Table 1. Comparison analyses between no-DC and DC groups before PSM

**Parameters**		**Enrolled patient (n = 443)**		**No-DC (n = 68)**		**DC (n = 375)**		**p-value**
Age		58.97	±17.716	66.38	±15.418	57.63	±17.791	**0.0002***
Sex (female)		116	(26.2%)	16	(23.5%)	100	(26.7%)	0.5886
Charlson comorbidity index		0.67	±1.254	0.97	±1.820	0.61	±1.115	0.0343*
Antiplatelet Medication		60	(13.5%)	8	(11.8%)	52	(13.9%)	0.6417
Alcohol Use		76	(17.2%)	11	(16.2%)	65	(17.3%)	0.8159
Mechanism	Unknown	64	(14.4%)	12	(17.6%)	52	(13.9%)	0.7715
	Fall	120	(27.1%)	15	(22.1%)	105	(28.0%)	
	TA Pedestrian	81	(18.3%)	14	(20.6%)	67	(17.9%)	
	TA Bicycle	58	(13.1%)	8	(11.8%)	50	(13.3%)	
	TA Motorcycle	31	(7.0%)	4	(5.9%)	27	(7.2%)	
	TA In-Car	38	(8.6%)	9	(13.2%)	29	(7.7%)	
	Assault	28	(6.3%)	3	(4.4%)	25	(6.7%)	
	Hit	1	(0.2%)	1	(1.5%)	0	(0.0%)	
	Sports	22	(5.0%)	2	(2.9%)	20	(5.3%)	
**Neurologic Status**								
Pupillary Response	Equal Reacting	162	(40.5%)	24	(37.5%)	138	(41.1%)	0.7854
	Unequal Reacting	21	(5.3%)	4	(6.3%)	17	(5.1%)	
	Only One Reacting	20	(5.0%)	2	(3.1%)	18	(5.4%)	
	Neither Reacting	197	(49.3%)	34	(53.1%)	163	(48.5%)	
Level Of Consciousness	Alert	25	(5.7%)	3	(4.5%)	22	(5.9%)	0.5283
	Drowsy	67	(15.2%)	8	(11.9%)	59	(15.8%)	
	Stupor	158	(35.8%)	23	(34.3%)	135	(36.1%)	
	Semicoma	144	(32.7%)	22	(32.8%)	122	(32.6%)	
	Coma	47	(10.7%)	11	(16.4%)	36	(9.6%)	
Glasgow Coma Scale score		7.80	±3.776	7.11	±3.534	7.92	±3.811	0.1732
**Vital Signs**								
NBP (mmHg)		103.18	±24.262	99.60	±30.436	103.83	±22.969	0.1875
SBP (mmHg)		144.05	±35.414	138.91	±46.612	144.97	±32.999	0.1971
DBP (mmHg)		82.73	±21.116	79.91	±23.873	83.24	±20.576	0.2341
HR		87.08	±23.380	91.96	±29.581	86.21	±22.026	0.0649
RR		19.24	±4.215	18.30	±5.710	19.41	±3.874	**0.0486***
BT ('c)		36.34	±0.957	36.16	±1.724	36.37	±0.746	0.1266
SpO_2 (%)		94.65	±7.789	92.39	±12.252	95.07	±6.598	**0.0188***
**Radiologic Features**								
Dx	EDH	30	(6.8%)	4	(5.9%)	26	(6.9%)	0.881
	SDH	299	(67.5%)	50	(73.5%)	249	(66.4%)	
	Contusion/ICH	59	(13.3%)	7	(10.3%)	52	(13.9%)	
	SAH, IVH	38	(8.6%)	7	(10.3%)	31	(8.3%)	
	Others	17	(3.8%)	0	(0.0%)	17	(4.5%)	
Rotterdam score		4.18	±1.338	4.13	±1.434	4.19	±1.321	0.7578
	1	3	(0.7%)	1	(1.5%)	2	(0.5%)	
	2	70	(15.8%)	12	(17.6%)	58	(15.5%)	
	3	60	(13.5%)	8	(11.8%)	52	(13.9%)	
	4	97	(21.9%)	18	(26.5%)	79	(21.1%)	
	5	138	(31.2%)	14	(20.6%)	124	(33.1%)	
	6	75	(16.9%)	15	(22.1%)	60	(16.0%)	
Basal Cistern	Collapse	98	(22.1%)	11	(16.2%)	87	(23.2%)	0.4406
	Compressed	137	(30.9%)	23	(33.8%)	114	(30.4%)	
	Preserved	208	(47.0%)	34	(50.0%)	174	(46.4%)	
Midline Shifting (>5 mm)		246	(55.5%)	39	(57.4%)	207	(55.2%)	0.7424
Midline Shifting (Mm)		7.77	±7.041	7.74	±7.141	7.78	±7.033	0.9671
Presence Of EDH (Mass Effect)		24	(5.4%)	4	(5.9%)	20	(5.3%)	0.8541
Presence Of SAH, IVH		190	(42.9%)	28	(41.2%)	162	(43.2%)	0.7564
Location	Left	146	(33.0%)	30	(44.1%)	116	(30.9%)	0.2223
	Right	212	(47.9%)	28	(41.2%)	184	(49.1%)	
	Bilateral (Or Diffuse)	83	(18.7%)	10	(14.7%)	73	(19.5%)	
**Notified Events**								
Vital Organ Damage		70	(15.8)	14	(20.6%)	56	(14.9%)	0.2412
Hypoxemia		64	(14.4)	3	(4.4%)	61	(16.3%)	0.0179
Shock		37	(8.4)	5	(7.4%)	32	(8.5%)	0.7464
Cpr		17	(3.8%)	5	(7.4%)	12	(3.2%)	0.111
**Laboratory Findings**								
Hb		12.902	±2.2619	12.844	±2.3617	12.913	±2.2465	0.8169
WBC		12704.63	±5650.937	11895.59	±5577.062	12851.33	±5659.194	0.1998
Plt		201.2483	±73.45181	205.3971	±76.40605	200.4960	±72.98347	0.6124
PT (sec)		13.31	±4.163	13.99	±5.410	13.18	±3.890	0.1505
aPTT (sec)		34.25	±28.831	33.51	±14.628	34.39	±30.728	0.8186
INR		1.1612	±0.39349	1.2265	±0.53802	1.1494	±0.36077	0.1486
Glucose		183.19	±73.055	185.60	±71.909	182.75	±73.348	0.7668
BUN		17.27	±10.123	15.44	±5.192	17.60	±10.750	0.0906
Creatinine		1.01	±1.057	±.93	±0.849	1.02	±1.091	0.5287
Sodium		139.63	±4.316	139.35	±4.384	139.67	±4.307	0.5712
Potassium		3.72	±0.585	3.68	±0.556	3.72	±0.590	0.5836
AST		75.06	±114.891	72.44	±101.913	75.54	±117.221	0.8379
ALT		42.52	±60.191	46.16	±95.129	41.86	±51.501	0.5896
Bilirubin		0.85	±2.115	0.82	±0.687	0.85	±2.281	0.896
Amylase		72.03	±71.718	68.93	±67.843	72.59	±72.473	0.7017
CRP		6.43	±24.900	7.40	±23.967	6.26	±25.090	0.733
