# Supplementary material for: The significance of decompressive craniectomy for older patients with traumatic brain injury: a propensity score matching analysis from large multi-center data
Source: Sci Rep. 2023 Jun 28;13:10498. doi: 10.1038/s41598-023-37283-x (PMC10307774; doi:10.1038/s41598-023-37283-x)
Supplement: Supplementary file 2 — Supplementary Table 2. [file 41598_2023_37283_MOESM2_ESM.pdf]

Supplementary Table 2. Comparison analyses between Young and Old TBI groups

Parameters		Young (n=269)		Old (n=174)		P-value
Age		48.36	±14.319	75.37	±6.222	**<0.001***
Sex (female)		72	26.8%	44	25.3%	0.730
Charlson comorbidity index		0.461	±0.990	0.983	±1.526	**<0.001***
Antiplatelet medication		37	13.8%	23	13.2%	0.872
Alcohol use		47	17.5%	29	16.7%	0.826
Mechanism	unknown	39	14.5%	25	14.4%	0.275
	fall down	79	29.4%	41	23.6%	
	TA pedestrian	49	18.2%	32	18.4%	
	TA bicycle	39	14.5%	19	10.9%	
	TA motorcycle	13	4.8%	18	10.3%	
	TA in-car	19	7.1%	19	10.9%	
	assault	16	5.9%	12	6.9%	
	hit	1	.4%	0	0.0%	
	sports	14	5.2%	8	4.6%	
**Neurologic status**						
Pupillary response	equal reacting	96	40.3%	66	40.7%	0.995
	unequal reacting	12	5.0%	9	5.6%	
	only one reacting	12	5.0%	8	4.9%	
	neither reacting	118	49.6%	79	48.8%	
Level of consciousness	alert	18	6.7%	7	4.0%	0.553
	drowsy	40	14.9%	27	15.6%	
	stupor	100	37.3%	58	33.5%	
	semicoma	81	30.2%	63	36.4%	
	coma	29	10.8%	18	10.4%	
Glasgow Coma Scale Score		8.01	±3.833	7.49	±3.684	0.242
**Vital signs**						
NBP (mmHg)		103.4774	±21.38380	102.7031	±28.13360	0.744
SBP (mmHg)		145.59	±32.847	141.70	±38.993	0.260
DBP (mmHg)		82.42	±18.032	83.21	±25.159	0.703
HR		86.88	±22.507	87.39	±24.741	0.821
RR		19.07	±4.211	19.49	±4.220	0.311
BT ('c)		36.421	±0.725	36.220	±1.2239	0.053
SpO2 (%)		95.31	±5.992	93.60	±9.944	**0.029***
**Radiologic features**						
Dx	EDH	19	7.1%	11	6.3%	0.249
	SDH	178	66.2%	121	69.5%	
	Contusion/ICH	43	16.0%	16	9.2%	
	SAH, IVH	20	7.4%	18	10.3%	
	others	9	3.3%	8	4.6%	
Rotterdam score		4.14	±1.349	4.24	±1.321	0.426
Basal cistern	Collapse	65	24.2%	33	19.0%	
	compressed	80	29.7%	57	32.8%	
	preserved	124	46.1%	84	48.3%	
Midline shifting (mm)		7.6521	7.05610	7.9693	7.03259	0.653
Location	Left	88	32.7%	58	33.3%	0.721
	Right	128	47.6%	84	48.3%	
	Bilateral (or diffuse)	51	19.0%	32	18.4%	
**Notified events**						
Vital organ damage		41	15.2%	29	16.7%	0.688
Hypoxemia		36	13.4%	28	16.1%	0.428
Shock		19	7.1%	18	10.3%	0.223
CPR		8	3.0%	9	5.2%	0.239
**Laboratory findings**						
Hb		12.991	±2.220	12.765	±2.325	0.304
WBC		12769.777	±5562.764	12603.908	±5799.359	0.763
Plt		199.108	±76.086	204.557	±69.268	0.446
PT_sec		13.421	±4.268	13.133	±4.001	0.478
aPTT_sec		35.519	±35.935	32.298	±10.937	0.252
INR		1.175	±0.411	1.139	±0.366	0.346

Glucose	185.657	±74.473	179.385	±70.859	0.378
BUN	16.744	±10.527	18.082	±9.436	0.175
Creatinine	0.967	±1.068	1.078	±1.040	0.281
Sodium	139.457	±4.279	139.885	±4.371	0.309
Potassium	3.695	±0.596	3.746	±0.568	0.373
AST	75.829	±103.027	73.866	±131.631	0.861
ALT	43.416	±64.899	41.128	±52.133	0.697
Bilirubin	0.927	±2.666	0.726	±0.585	0.331
Amylase	69.414	±68.670	76.196	±76.354	0.337
CRP	6.587	±24.865	6.197	±25.026	0.873
**Outcome parameters**					
death_2wk	113	42.0%	80	46.0%	0.411
death_1mo	130	48.3%	89	51.1%	0.562
death_6mo	139	51.7%	97	55.7%	0.401
mRS_6mo	4.8	±1.709	4.89	±1.619	0.599
0	5	1.9%	1	.6%	0.450
1	22	8.2%	11	6.3%	
2	14	5.2%	12	6.9%	
3	7	2.6%	9	5.2%	
4	24	8.9%	14	8.0%	
5	58	21.6%	30	17.2%	
6	139	51.7%	97	55.7%	
Favorable mRS_6mo (0-3)	48	17.8%	33	19.0%	0.765
